# Comment on “A Pragmatic Approach to Resolving Technological Unfairness: The Case of Nike’s Vaporfly and Alphafly Running Footwear”

**DOI:** 10.1186/s40798-021-00378-8

**Published:** 2021-12-17

**Authors:** Víctor Rodrigo-Carranza, Fernando González-Mohíno, Jordan Santos-Concejero, José María González-Ravé

**Affiliations:** 1 Sport Training Lab, University of Castilla-La Mancha, Toledo, Spain; 2 Facultad de Ciencias de la Vida y de la Naturaleza, Universidad Nebrija, Madrid, Spain; 3 Department of Physical Education and Sport, University of the Basque Country UPV/EHU, Vitoria‑Gasteiz, Spain

**To the Editor,**

We have read with great interest the manuscript recently published in your journal on the technological unfairness of the use of Nike Vaporfly and Alphafly running footwear models [1]. In the section “*Do the Nike Vaporfly/Alphafly Shoes Provide an Unfair Advantage?”,* the author claimed that the performance progression in long-distance, such as the marathon and the half-marathon, has not changed and that *“the shoes’ introduction has not obtained any greater change than the reasons attributed to any former record being broken”.*

## Background

Joyner et al. [2] evaluated the progression of world record performances in marathon since the late 1920s. On average, a 20s reduction per year since 1960 was found, but since 1980, the average reduction was lower (10s) per year, showing a stagnation in progression. Since the release of this type of footwear (2017), all men’s and women’s world records in long-distance road running events have been broken by athletes wearing new technological footwear [3]. Moreover, it has been suggested that *“recent improvements in these events are unlikely physiological but rather technological, if for no other reason that such a step-wise improvement in physiological attributes underpinning performance is unlikely”*. A recent meta-analysis [4] confirms that using shoes with increased longitudinal bending stiffness and a curved plate (such as the Vaporfly shoes) implies a technological improvement of ~ 3.45% in running economy (RE). These RE changes provide a ~ 2% improvement in marathon performance [5]. Therefore, the relation between RE improvement and performance is clear.

## Performance Enhancements in the New Technological Footwear Era

However, recent retrospective studies [6, 7] analysing the use of Vaporfly shoes in long-distance running events have confirmed improvements on the performance previously found in laboratory conditions. Bermon et al. [6] reported a decrease in elite athlete’s season best times in 10-km, half-marathon and marathon events from 2017 in the top 20 and top 100 best times per year. While this study assessed the footwear worn by runners only for the top 20 from 2016 to 2019, a recent study from our research group [8] identified more than the 90% of footwear worn in these three events for the top 100 best times per year from 2015 to 2019. For example, in the years before the 2017 (when this type of footwear was released), the year-on-year improvement of marathon performances between 2015 and 2017 was only of 0.14–0.21% for the top 100 best times, while from 2017 onwards the performance improved between 0.75 and 1.50% compared to 2015 and 2016. In addition, there was a strong correlation between number of runners using this footwear per year and marathon performance improvements (*r* = 0.96).

Moreover, another study from our research group (under review) evaluated the impact of the Vaporfly shoes on the best Marathon times in history, where 23 of the 50 (46%) best times in history (accessed 20 November 2020) have been achieved with Vaporflys, and 21 of them being achieved since 2018 (42%). In addition, from the best performances under 2:03:00, every performance except the one by Dennis Kimetto (former World Record, Berlin Marathon, 28 September 2014) has been achieved with Vaporfly shoes.

## Conclusion

We conclude that the new technological footwear implies a clear impact in long-distance running performance, and probably an unfair advantage due to the greater improvements they provide when compared to the years prior of the technological revolution.

## Data Availability

Not applicable.
